# Development of Multicenter Deep Learning Models for Predicting Surgical Complexity and Surgical Site Infection in Abdominal Wall Reconstruction, a Pilot Study

**DOI:** 10.3389/jaws.2025.14371

**Published:** 2025-04-14

**Authors:** William R. Lorenz, Alexis M. Holland, Benjamin A. Sarac, Samantha W. Kerr, Hadley H. Wilson, Sullivan A. Ayuso, Keith Murphy, Gregory T. Scarola, Brittany S. Mead, B. Todd Heniford, Jeffrey E. Janis

**Affiliations:** ^1^ Division of Gastrointestinal and Minimally Invasive Surgery, Department of Surgery, Carolinas Medical Center, Charlotte, NC, United States; ^2^ Department of Plastic and Reconstructive Surgery, Ohio State University Wexner Medical Center, Columbus, OH, United States; ^3^ Department of Computer Science, University of North Carolina at Charlotte, Charlotte, NC, United States

**Keywords:** artificial intelligence, ventral hernia repair, quality improvement, prediction model, component separation, deep learning model

## Abstract

**Objective:**

Hernia recurrence and surgical site infection (SSI) are grave complications in Abdominal Wall Reconstruction (AWR). This study aimed to develop multicenter deep learning models (DLMs) developed for predicting surgical complexity, using Component Separation Technique (CST) as a surrogate, and the risk of surgical site infections (SSI) in AWR, using preoperative computed tomography (CT) images.

**Methods:**

Multicenter models were created using deidentified CT images from two tertiary AWR centers. The models were developed with ResNet-18 architecture. Model performance was reported as accuracy and AUC.

**Results:**

The CST model underperformed with an AUC of 0.569, while the SSI model exhibited strong performance with an AUC of 0.898.

**Conclusion:**

The study demonstrated the successful development of a multicenter DLM for SSI prediction in AWR, highlighting the impact of patient factors over surgical practice variability in predicting SSIs with DLMs. The CST model’s prediction remained challenging, which we hypothesize reflects the subjective nature of surgical decisions and varying institutional practices. Our findings underscore the potential of AI-enhanced surgical risk calculators to risk stratify patients and potentially improve patient outcomes.

## Introduction

Recent advances in artificial intelligence (AI) have demonstrated remarkable capabilities in the diagnosis and characterization of pathologies through computed tomography (CT) images, underscoring its potential as an indispensable tool in the surgical decision-making process [1–4]. Particularly in abdominal wall reconstruction (AWR), AI’s predictive power promises to enhance operative planning and patient counseling, thus potentially improving the overall quality of care. In prior research on AWR, our team successfully developed and internally validated image-based deep learning models (DLMs) designed to anticipate the level of surgical complexity and the risk of surgical site infections (SSI) [5]. This innovation was the first of its kind, utilizing preoperative CT imaging to foresee the likelihood of requiring a component separation technique (CST), which is a proxy for operative complexity, and predicting surgical site infection (SSI).

The AI model’s proficiency in drawing from preoperative imaging to predict intraoperative events and postoperative outcomes signals a leap toward personalized surgical risk assessment and precision medicine that has been lacking in the field [1, 2, 6, 7]. First, AI in AWR will help surgeons identify patients who are at risk for a complex surgical operation in addition to postoperative complications. Successful implementation of such a model will allow appropriate triage of the patient to the proper surgeon, whether that is local to them, or at a tertiary hernia center. Additionally, the surgeon will be able to evaluate each patient’s preoperative risk of complications, including SSI, and therefore be better able to counsel patients, obtain preoperative optimization, and prepare for intraoperative decision making. Particularly in AWR, this means accomplishing a low recurrence rate and low rate of postoperative surgical site occurences. Achieving these outcomes not only benefits the patient but also the hospital system as a whole [8]. The financial cost of complications in AWR is staggering, and reducing recurrence rates by 1% was estimated to save $139.9 million annually [8–10]. Given the annual incidence of around 611,000 AWR cases, optimization of outcomes has the potential to greatly reduce hospital resource utilization in the United States [9–12]. As previously discussed, the push for establishing AWR tertiary centers is ongoing [13–16], but empowering community general surgeons and equipping specialists alike with tools to optimize outcomes will have far reaching benefits.

The true test of any AI-based model’s utility and generalizability lies in its ability to obtain external validity [17]. This is the foundation to evaluate the transferability and reliability of the DLMs predictions to external cohorts and ensures that the models perform well when confronted with the variability inherent to different surgical practices and patient populations [7]. Therefore, the aim of the current study was to construct a multicenter model and test its performance.

## Methods

### Study Design

Study design and result reporting were based on the Transparent Reporting of a Multivariable Prediction Model for Individual Prognosis or Diagnosis (TRIPOD) reporting guidelines [18]. With institutional review board approval and a joint data sharing agreement, a multicenter DLM was developed. One center used the original CST and SSI images employed by Elhage et al [5] in the development of an internally validated model. The other center’s images were obtained from a cohort of 75 patients, who were treated by an AWR specialist at a tertiary center in a different region of the United States. Both patient groups underwent preoptimization including smoking cessation for a minimum of 4 weeks, preoperative weight-loss, and reduction of HgbA1c to less than 7.2 mg/dL [19, 20]. Patients whose CT scans with scatter (secondary to orthopedic prosthetics, for example,) that limited the algorithm’s interpretation of the image were excluded from model training. Additionally, those who had a chemical component relaxation with botulinum toxin A injection were excluded, as this would alter the rate of CST performed on large, loss of domain, hernias. A CST was either an anterior or poster myofascial release that was either unilateral or bilateral. CST technique and algorithm varied between institutions [21, 22]. Both institutions perform a step-up approach of an anterior or posterior CST. The patients were reported as having a CST if any portion of the CST procedure was performed, even if a full musculofascial release was not performed. SSI was defined as a deep or superficial wound infection. A deep infection included a deep space or mesh infection, whereas a superficial infection included a subcutaneous infection or cellulitis [23].

### Development and Validation of DLM

CST and SSI prediction models were built from the original internal dataset with the established ResNet-18 architecture using PyTorch software version 1.13.1 [24]. The model architecture is comprised of 18 unique layers that include the initial convolutional layer, four sets of four convolutional layers of similar filter size, and finally a fully connected layer. ResNet-18 architecture uses the stochastic gradient descent optimizer and the sparse binary cross-entropy loss function for model training [25]. Finally, transfer learning was performed using pretrained model weights for ResNet-18 on the ImageNet database.

Model consistency was assessed using Leave-One-Out Cross-Validation (LOOCV) and k-fold cross-validation across multiple training runs, which provides less biased assessment than the traditional test:train split [26]. Specifically, LOOCV involves a series of training runs that equals the number of events. The model sequentially leaves one event out, trains the model on the other events, and tests the newly trained model on the left-out event. This is repeated until all events are tested. The results of the predictions are then averaged. This was performed for the CST and SSI models separately.

### DLM Predictions and Evaluation

Statistical analysis was performed using Python version 3.7.1 by a data scientist. For internal validation, an 80:20 train:validation split was used. The models were assessed for discernibility and compared by training and validation accuracy, as well as the validation AUC score, across five training runs [27].

## Results

### Cohort Description

The internal CST sample had 297 patients (97 underwent CST). The internal SSI sample had 362 patients (77 with an SSI). The external cohort had 75 patients. Of which, 48 patients underwent CST, and 13 patients developed an SSI.

### Leave-One-Out Cross-Validation

To build the DLMs with the ResNet-18 Architecture, the patients were divided into cohorts CST and SSI as described. LOOCV revealed that both models showed good performance. The CST model had an overall classification accuracy of 75% of cases. SSI performed better with 94.65% accuracy across the dataset.

### Pooled Multicenter Cross-Validation

The internal and external combined cohort had 297 patients in the CST model and 362 patients in the SSI model. The CST model consisted of 237 internal patients and 60 external patients, with 77 and 38 CSTs in each group, respectively. The SSI model consisted of 300 internal patients and 62 external patients, with 64 and 11 SSIs in each group, respectively (Table 1).

**TABLE 1:** Cohort data.

	Overal	Internal Patients	External Patients
CST Sample	297	237 (79.8%)	60 (20.2%)
CST Yes^a^	115	77 (67.0%)	38 (33.0%)
SSI Sample	362	300 (82.9%)	62 (17.1%)
SSI Yes^a^	75	64 (85.3%)	11 (14.7%)

^a^ Patients who required CST or developed SSI of the entire cohort of images reviewed.

Note: CST, component separation technique; SSI: surgical site infection. Data are presented as n(%).

**FIGURE 1:**
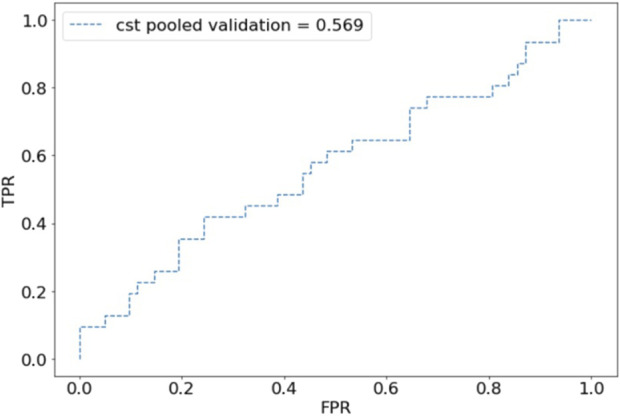
Receiver operating characteristic (ROC) plot for component separation technique (CST) predictions of pooled validation group.

**FIGURE 2:**
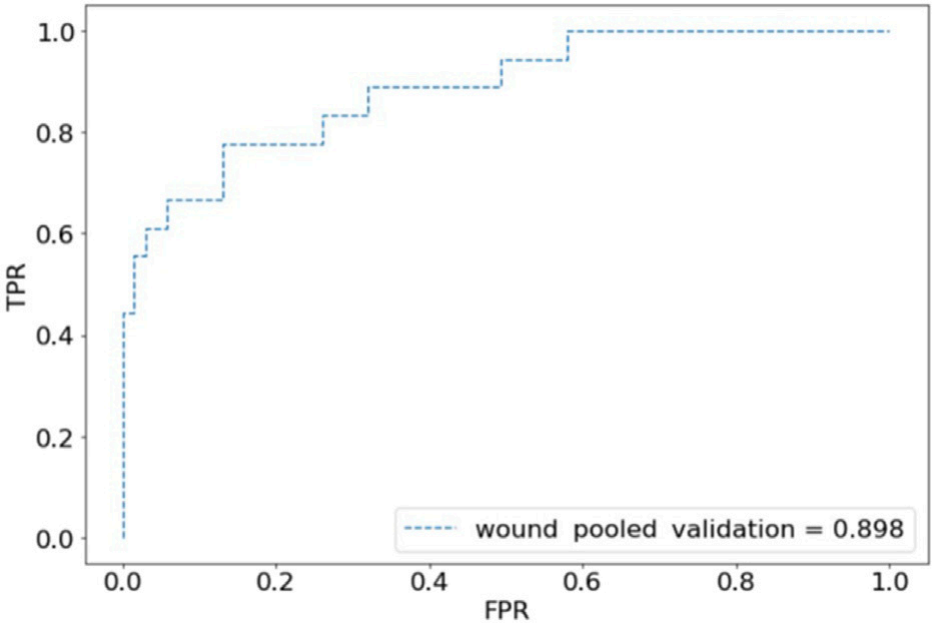
Receiver operating characteristic (ROC) plot for wound complications predictions of pooled validation group.

For internal validation, after an 80:20 train:test split, the CST pooled cohort had training accuracy of 91.26%, validation accuracy of 39.53%, and an AUC of 0.569 (Figure 1). the sensitivity was 41.94% and specificity of 67.77%. The SSI performed better with training accuracy of 97.92%, validation accuracy of 88.61%, AUC of 0.898 (Figure 2), sensitivity of 55.56%, and specificity of 95.65%.

## Discussion

This study describes the first known efforts to create and validate multicenter DLMs using AI to predict surgical complexity and postoperative outcomes. The results show proof of concept for multicenter development of image-based DLMs. While we have previously developed and demonstrated DLMs’ ability to predict intraoperative and postoperative outcomes, external validation has not been performed [5, 28]. A multicenter model was developed to evaluate whether pooled training and analysis would improve the models’ performance. While the CST model showed poor performance with a validation accuracy of 39.53% and an AUC of 0.568, the SSI model was more promising with a validation accuracy of 88.61% and an AUC of 0.879.

In general, external validation of predictive models is rarely described in the literature with only 5% of the approximately 85,000 prediction model publications on PubMed including some form of external validation [17, 29]. Specifically, many commonly used AWR risk stratification tools lack external validation [7]. To temper the recent excitement of using AI in surgical decision-making, Loftus et al recently called for more rigorous external validation, especially for AI prediction models [1]. This study was conducted to help address this evident gap in the literature.

Creating an externally validated DLM has many benefits, namely, its ability to become an advanced surgical risk calculator to provide personalized and informed patient counseling. There are currently several surgical risk calculators for AWR [7, 30]. The group at Carolinas Medical Center has previously published work aimed at predicting outcomes and patient centered care through the Carolinas Equation for Determining Associated Risk (CeDAR) application, which identifies patients that are at risk of wound complications after AWR along with their predicted costs [7, 31]. Unlike DLMs, this app requires human input to estimate risk [31]. Our group has also used volumetric assessment of CT scans to estimate surgical risk [32, 33]. The limitation to this method is the time and labor involved, as well as the subjectivity in data collection. DLMs can improve a surgeon’s predictive ability and aid in surgical planning and patient counseling [1, 3]. The end goal of DLMs is not to replace a surgeon’s clinical judgment, but rather augment it [1, 2].

The CST model performed poorly. While achieving fascial closure is the goal in AWR, techniques to achieve this vary [21, 34, 35]. The decision to perform a CST is complex and subjective, and practices often differ from institution to institution as well as patient to patient [21, 34]. There is a difference in practice and patient population, between the institutions, which is evident in the frequency of CST in each cohort [21]. While the authors attempted to propensity match the internal and external groups, this further limited the sample size. Therefore, the decision was made to continue without propensity matching. As a result though, differences in patient factors, such as hernia size or BMI, could contribute to the differences in rate of component separation. Another potential contributor to the poor performance of the CST model is the inability to predict tissue compliance. Past medical history and imaging do not capture compliance, as it is a difficult component to measure, but we suspect this too played a role in the model’s performance.

Additionally, CST is a broad term that can be used for many specific procedures. While some surgeons may choose to do a posterior component separation, or Transversus Abdominus Release (TAR), others may choose an anterior approach. While both techniques have their advantages, individual patient differences may lead a surgeon to perform one technique over the other [21, 22, 34]. The surgeons of the internal cohort choose to perform an anterior or posterior CST based on defect size [21]. The surgeon of the external cohort also performs both anterior and posterior CST, but typically performs anterior CST for larger defects. Given the varied practice patterns, it is difficult to train a reliable and predictive model that will perform on external data [17, 29]. Even with pooled training and analysis the poor performance of the model is likely explained by the nuanced practice difference between AWR centers.

On the other hand, the SSI model was found to have excellent predictive ability. An explanation for this finding may be that patient factors such as obesity and predisposing comorbidities, rather than institutional differences in surgical practice, are more likely determinants of developing SSIs [8, 9, 20, 36, 37]. Factors such as the amount of subcutaneous adipose tissue, as a surrogate for BMI, are evident on the CT scans and may contribute to the model’s ability to predict outcomes [32, 33, 38–42]. Predicting and preventing SSIs is vital for successful AWR. SSIs have been shown to increase a patient’s risk of developing a hernia recurrence by three to five times [8, 43, 44]. Additionally, superficial wound complications increase a patient’s likelihood of a mesh infection, which is a feared complication of AWR, that will likely lead to further operations in the future [43, 45].

Not only are SSIs responsible for poor patient outcomes, but also for increased healthcare spending [8, 9, 11]. The cost of complications has been explored in prior work [9]. The difference in outpatient charges between patients with and without a complication is $6,200 ± 13,800 and $1,400 ± 7,900, respectively, with more than four more office visits [9]. Determining which patients are at an increased risk for postoperative wound complications allows surgeons to intervene and decrease the risk of complications. Optimization of patients’ outcomes could either be preoperative, in the form of preoptimization, intraoperative, or postoperative. Intraoperatively, maintaining strict sterility, judicious handling of the skin and soft tissues, as well as electing to use closing protocols can decrease the rate of SSI [20, 37, 46]. Postoperative options include the decision to perform a delayed primary closure (DPC) or apply a closed incision negative pressure wound therapy vacuum [19, 47, 48].

This study is not without limitations. A pooled multicenter analysis was performed, yet again, the CST model did not perform well. An explanation for the initial model’s poor performance is the skewed nature of the datasets. The external cohort was limited with 75 patients. The external cohort also had different proportions of CST procedures performed. This is due to different AWR practice models. The internal group often uses botulinum toxin injections as a means to prevent the need for CST. This may differ from the practice algorithm of the external validation group or even other practices that may use techniques such as progressive pneumoperitoneum. This inherently is a limitation with comparing different medical centers and practices and may make our study less generalizable. Further, models developed with ResNet-18 are known to perform better with skewed data sets, like this study. Knowing the skewed nature of the datasets allows the model to be scaled appropriately. While training and validating a model based on pooled data seems promising, it is likely that a multi-institution model would need to be developed to account for the vast difference in practice patterns in CST among AWR surgeons.

This study is the first of its kind demonstrating techniques to externally validate a predictive surgical model. We demonstrated that while CST is challenging to predict, the SSI model performed well in a multicenter setting. This study indicates that models can predict outcomes where patient factors are readily evident in the data but are limited where there is subjectivity in surgical management. Future directions for study should look to train AI models on large multicenter databases to account for variations in surgical practice.

## Data Availability

The raw data supporting the conclusions of this article will be made available by the authors, without undue reservation.
